# Neuromuscular Junction Defects in Mice with Mutation of *dynein heavy chain 1*

**DOI:** 10.1371/journal.pone.0016753

**Published:** 2011-02-04

**Authors:** Stephanie L. Courchesne, Maria F. Pazyra-Murphy, Daniel J. Lee, Rosalind A. Segal

**Affiliations:** 1 Departments of Cancer Biology and Pediatric Oncology, Dana Farber Cancer Institute, Boston, Massachusetts, United States of America; 2 Department of Neurobiology, Harvard Medical School, Boston, Massachusetts, United States of America; Tokyo Medical and Dental University, Japan

## Abstract

Disruptions in axonal transport have been implicated in a wide range of neurodegenerative diseases. *Cramping 1* (*Cra1*/+) and *Legs at odd angles* (*Loa*/+) mice, with hypomorphic mutations in the *dynein heavy chain 1* gene, which encodes the ATPase of the retrograde motor protein dynein, were originally reported to exhibit late onset motor neuron disease. Subsequent, conflicting reports suggested that sensory neuron disease without motor neuron loss underlies the phenotypes of *Cra1*/+ and *Loa*/+ mice. Here, we present behavioral and anatomical analyses of *Cra1*/+ mice. We demonstrate that *Cra1*/+ mice exhibit early onset, stable behavioral deficits, including abnormal hindlimb posturing and decreased grip strength. These deficits do not progress through 24 months of age. No significant loss of primary motor neurons or dorsal root ganglia sensory neurons was observed at ages where the mice exhibited clear symptomatology. Instead, there is a decrease in complexity of neuromuscular junctions. These results indicate that disruption of dynein function in *Cra1*/+ mice results in abnormal morphology of neuromuscular junctions. The time course of behavioral deficits, as well as the nature of the morphological defects in neuromuscular junctions, suggests that disruption of dynein function in *Cra1*/+ mice causes a developmental defect in synapse assembly or stabilization.

## Introduction

Rapid axonal transport is essential for neurons with long axons, both for development and mature functioning. Without transport, neurotransmitter vesicles cannot move to synapses, damaged organelles in the axon cannot be replaced by healthy organelles, and target-derived survival signals cannot reach the cell body. Because of the importance of axonal transport, it is not surprising that disruptions in this process have been implicated in a wide range of neurodegenerative diseases, including motor and sensory neuropathies, Alzheimer's disease, Parkinson's disease, and Huntington's disease [1]–[4]. Perhaps because of the long distance between cell bodies and distal axons in these neurons (over 1 meter in an adult human), sensory and motor neurons appear to be particularly sensitive to disruptions in axonal transport.

Disruptions in retrograde transport have been implicated in neurodegenerative disease in mice as well as in humans. Disrupting the function of the retrograde motor dynein in motor neurons by overexpression of dynamitin causes late-onset motor neuron degeneration in mice [5], [6]. A mutation in the p150^Glued^ subunit of the dynactin complex has been discovered in a family with a progressive autosomal dominant form of lower motor neuron disease, and mice with mutations in p150^Glued^ develop motor neuron disease [7], [8], [6].

In 2003, Fisher and colleagues reported two lines of mice with late onset, progressive motor neuron disease, called *Legs at odd angles (Loa*/+) and *Cramping 1 (Cra1*/+) [9]. Both *Loa*/+ and *Cra1*/+ mice have a missense mutation in the *dynein heavy chain 1* gene [9]. Heterozygous mice from both lines were reported to show an age-related, progressive motor neuron disease, with cramping of hindlimbs, gait abnormalities, and eventual paralysis. While the number of motor neurons was normal in *Cra1*/+ mice at three months of age, a severe reduction in motor neuron number was reported at 16 months of age in *Cra1*/+ mice and 18 months of age in *Loa*/+ mice, consistent with the progressive symptoms. *In vitro*, neurons from *Loa/Loa* embryos exhibited a defect in the fast component of retrograde transport without other alterations in the ubiquitous functions of dynein, such as nuclear motility and Golgi complex morphology and positioning. Furthermore, purified dynein from *Loa/+* and *Loa/Loa* mice exhibited reduced processivity, premature run termination and altered coordination of motor domains. These defects together lead to impaired retrograde axonal transport [10]. Thus, it was concluded that neuron degeneration in these mice arises from specific disruptions in rapid retrograde axonal transport [9], [10].

In contrast, two recent studies provide evidence that the *Loa*/+ and *Cra1*/+ mice exhibit a sensory neuropathy without motor neuron involvement. Electromyography recordings, motor neurons counts, and neuromuscular junction analysis in aged (18–22 months) *Loa*/+ and *Cra1*/+ mice all demonstrate normal motor neuron numbers and innervation of muscle [11], [12]. Both lines of mice were reported to exhibit an early-onset sensory neuropathy, with reduced numbers of large caliber proprioceptive axons in the dorsal root and reduced innervation of muscles spindles. These results are similar to the phenotype reported for Sprawling (*Swl*/+) mice, another hypomorphic mutation in *dynein heavy chain 1* that exhibits proprioceptive deficits and loss of proprioceptive neurons [13].

In order to clarify the link between disruption in axonal transport and neuronal functioning we undertook a detailed examination of the behavioral and anatomic phenotype in *Cra1*/+ mice. Behavioral analysis revealed an early onset, stable phenotype in *Cra1*/+ mice, with hindlimb cramping and impaired grip strength. We find no evidence of motor or sensory neuron loss in the *Cra1*/+ mice at symptomatic ages. Strikingly however, neuromuscular junctions in *Cra1/+* mice are abnormal. While the number and size of these synaptic zones are unchanged, the complexity of the pretzel-like NMJ is significantly reduced in mutant animals. Furthermore, analysis of muscle fibers provides evidence of continuous muscle degeneration and regeneration. These results suggest that the primary pathology in *Cra1/+* animals may be an early onset, non-progressive synaptic dysfunction that affects the neuromuscular junction.

## Results

### *Cra1*/+ Mice Show an Early Onset, Stable Phenotype of Neuronal Dysfunction

**Figure 1:**
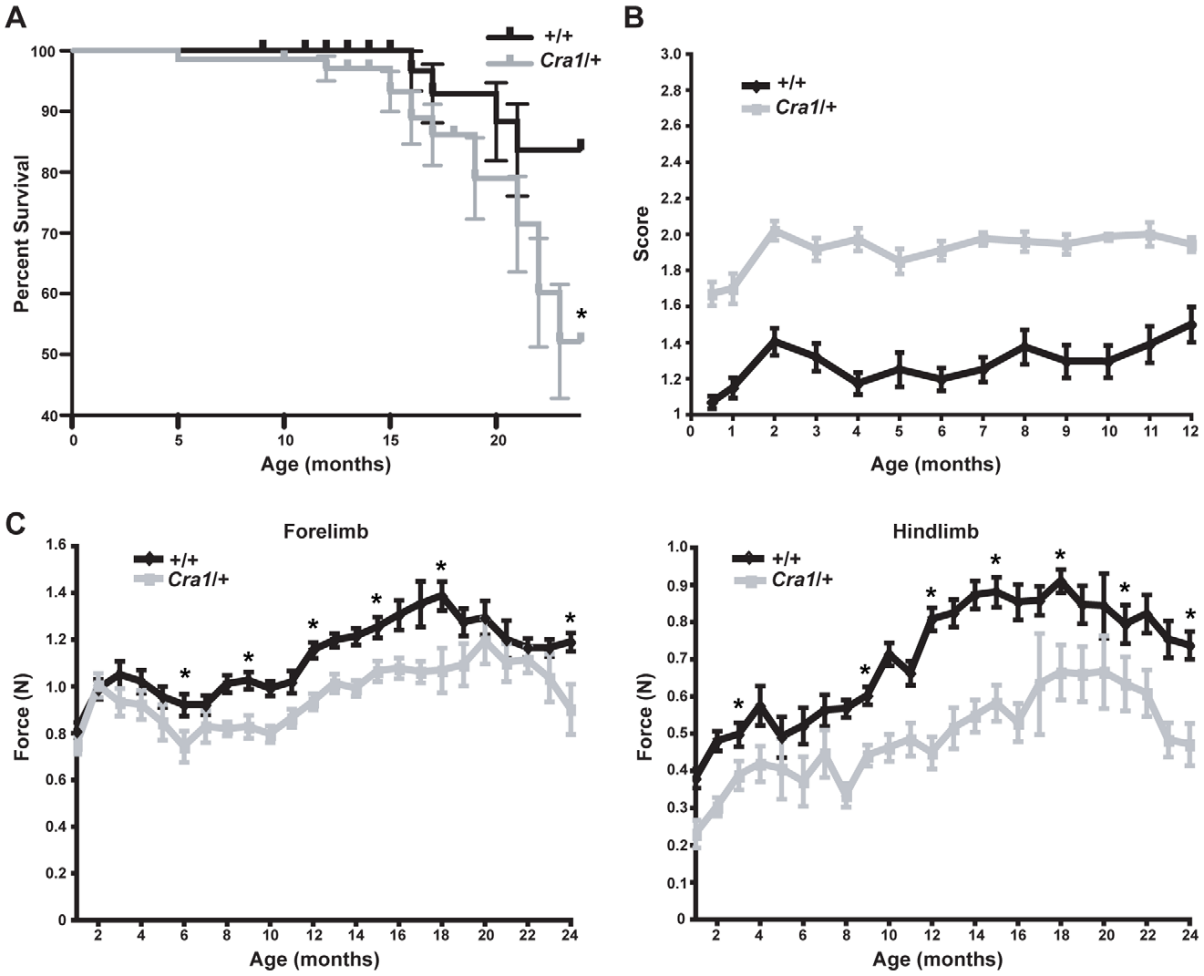
*Cra1*/+ mice show early-onset, non-progressive cramping of hindlimbs and reduced grip strength in fore- and hindlimbs. A) Kaplan-Meier survival curves show a significant difference in survival between +/+ and *Cra1*/+ mice (*P<0.05). Cause of death is unknown, but there was no evidence of nervous system dysfunction. B) Analysis of the “cramping” posture of +/+ and *Cra1*/+ mice. Quantification of average cramping scores for +/+ and *Cra1*/+ mice from 15 days old through 12 months old shows an early onset, stable phenotype of increased cramping posture in the *Cra1*/+ mice (N = 17–34 +/+; N = 19–36 *Cra1*/+). C) Grip strength measurements for both fore- and hindlimb show significant impairments in grip strength for *Cra1*/+ mice beginning at early ages. Grip strength in *Cra1*/+ animals does not decline over time (*P<0.05; comparisons between +/+ and *Cra1*/+ mice were calculated at 3 month intervals; N = 5–34 +/+; N = 5–31 *Cra1*/+).

*Cra1*/+ mice were originally reported to exhibit late onset, progressive degeneration of motor neurons [9]. In order to characterize disease progression in these mice, we undertook a long-term analysis of *Cra1*/+ mice. We find that *Cra1*/+ mice show reduced survival compared to wild-type (+/+) mice (Figure 1A). The cause of death was not evident in the majority of cases. We did not observe hindlimb paralysis, increased seizure activity, or other signs of nervous system or motor neuron dysfunction in the majority of animals that died prematurely. The original identification of the *Cra1*/+ line of mice was a result of their unusual “cramping” posture of the hindlimbs when suspended by the tail. We documented the emergence of this cramping posture in *Cra1*/+ mice between the ages of 15 days old and 12 months old by scoring animals as either 1 (no hindlimb abnormalities), 2 (cramping of hindlimbs), or 3 (paralysis of one or both hindlimbs). Surprisingly, we find that *Cra1*/+ mice demonstrate pronounced cramping posture as early as 15 days of age, the earliest time that could be tested (Figure 1B).

In order to assess motor neuron function in a quantitative fashion in *Cra1*/+ mice, we used a digital grip strength meter to record the power used to resist an opposing force with either fore- or hindlimbs. This behavioral test requires the mice to grip a wire grid attached to a force gauge and maintain their grip while they are pulled away from the grid, necessitating both proper gripping with the fore- and hindfeet as well as pulling with the limb muscles. Measurement of both fore- and hindlimb grip strength shows an early onset, stable reduction in grip strength compared to +/+ animals (Figure 1C). Interestingly, *Cra1*/+ mice show stable grip strength measurements over time, indicating that the dysfunction in grip strength does not progress with age.

### *Cra1*/+ Mice Do Not Show Evidence of Motor or Sensory Neuron Loss at Symptomatic Ages

**Figure 2:**
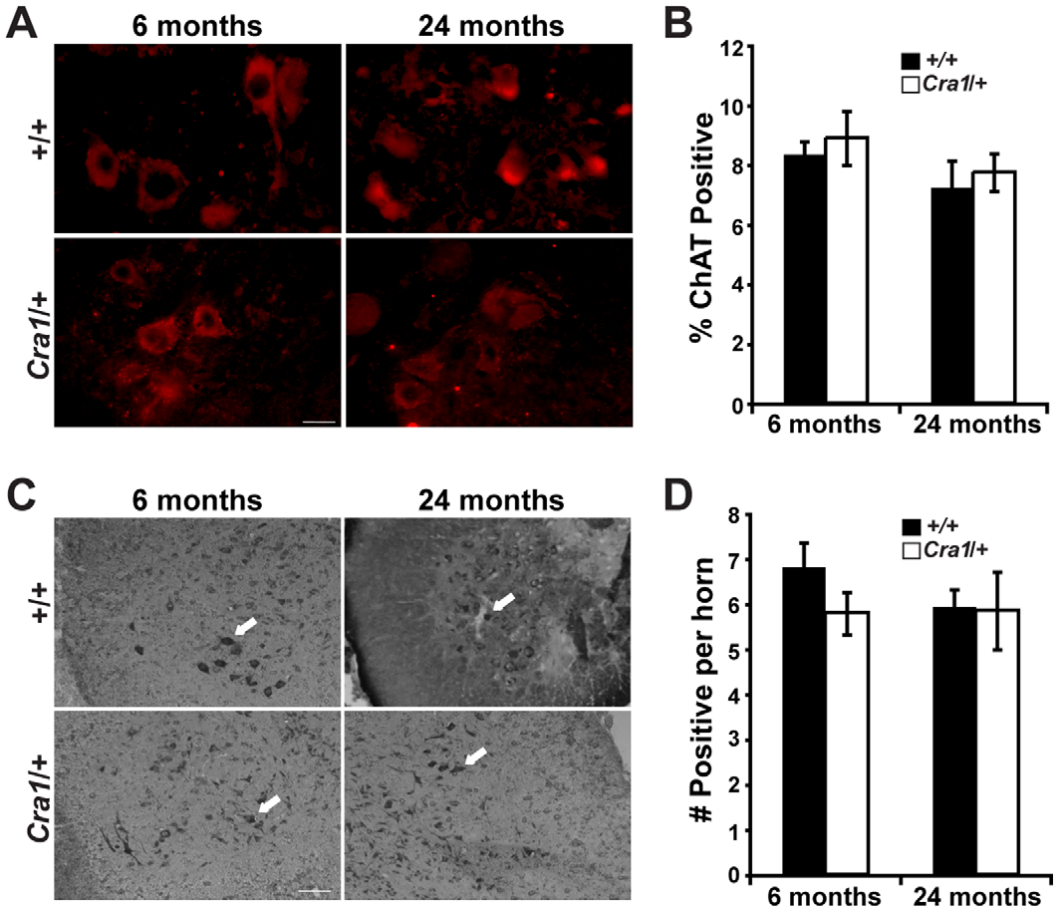
*Cra1*/+ mice show no evidence of motor neuron loss at symptomatic ages. A) Motor neuron labeling with ChAT of +/+ and *Cra1*/+ mice at 6 months and 24 months of age (Scale bar  = 50 µm). B) Quantification of ChAT-labeled motor neurons reveals no difference in number between +/+ and *Cra1*/+ mice (N = 3 animals per genotype). C) Toluidine blue staining of the anterior horn of the spinal cord at 6 months and 24 months of age in +/+ and *Cra1/+* mice (white arrows indicate motor neurons, Scale bar  = 50 µm). D) Quantification of toluidine blue labeled motor neurons reveals no difference in number between +/+ and *Cra1*/+ mice (N = 3 animals per genotype).

To investigate motor neuron degeneration in *Cra1*/+ mice, we examined motor neuron cell bodies in the spinal cord for evidence of pathology. Choline acetyltransferase (ChAT) labeling of motor neuron cell bodies in the ventral horn of the lumbar spinal cord in 6 and 24 month old *Cra1*/+ and +/+ mice demonstrates no observable difference in motor neuron morphology or ChAT expression in *Cra1*/+ mice (Figure 2A). Quantification of ChAT labeled motor neurons reveals no difference in neuronal number between *Cra1*/+ and +/+ mice (Figure 2B). The overall appearance and number of anterior horn motor neurons is also normal in 6 and 24 month old *Cra1/+* mice as observed by standard histochemistry with toluidine blue (Figure 2C, 2D). As 6 months is long after the emergence of abnormal hindlimb posture (15 days of age) and impairments in hindlimb grip strength (3 months of age), this result indicates that the observed neuronal dysfunction is not a result of motor neuron cell body loss.

**Figure 3:**
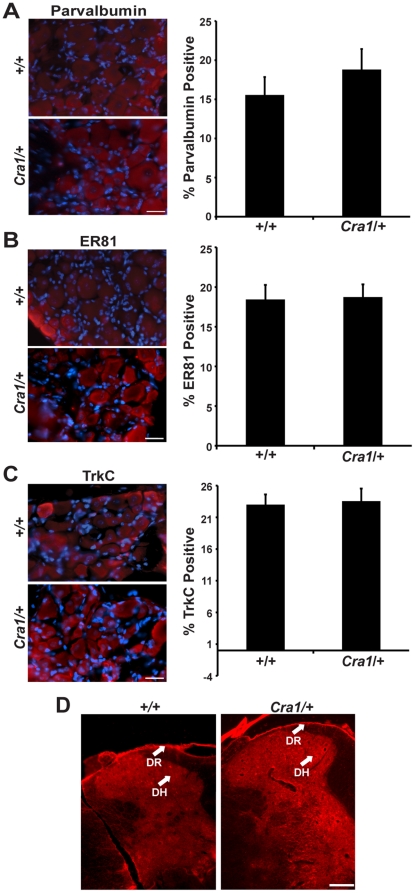
*Cra1*/+ mice show no evidence of proprioceptive sensory neuron loss at symptomatic ages. A) Proprioceptive sensory neuron labeling and quantification with parvalbumin, (B) ER81, and (C) TrkC shows no difference between of +/+ and *Cra1*/+ mice at 6 months of age (Scale bar  = 20 µm). D) Parvalbumin labeling of proprioceptive sensory neuron fibers within the spinal cord shows the central projection of these sensory neurons is intact in *Cra1*/+ mice (Scale bar  = 20 µm; N = 3 animals per genotype).

Recent studies suggested that neurologic deficits in *Cra1*/+ and *Loa*/+ mice are instead due to loss of proprioceptive sensory neurons [11], [12], a defect that would also result in the observed phenotypes of hindlimb cramping and reduced grip strength [14]. Using several markers for DRG proprioceptive neurons, including parvalbumin, ER81, and TrkC [15], [16], we observe no difference in proprioceptive neuron morphology or number between *Cra1*/+ and +/+ mice at 6 months of age (Figure 3A, 3B, 3C, respectively). Additionally, we examined the connection between proprioceptive afferents and primary motor neurons within the spinal cord. Labeling of these fibers in the spinal cord with parvalbumin revealed that proprioceptive fibers innervate motor neurons in the spinal cord to a similar extent in both *Cra1*/+ and +/+ mice (Figure 3D). Thus, proprioceptive neuron cell bodies, as well as their central projections, are intact in symptomatic *Cra1*/+ mice.

### *Cra1*/+ Mice Exhibit Reduced Complexity of Neuromuscular Junctions

**Figure 4:**
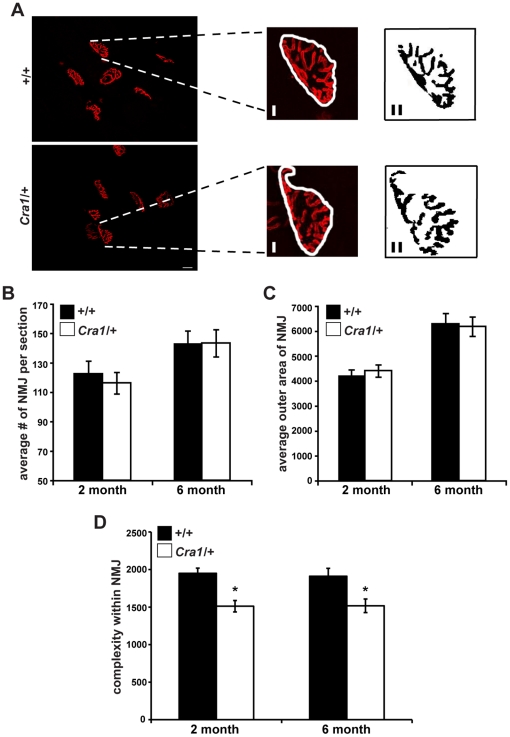
Neuromuscular junctions of *Cra1*/+ mice are abnormal at symptomatic ages. A) α-bungarotoxin labeled NMJs (Scale bar  = 20 µm) B) Quantification of α-bungarotoxin labeling demonstrates no differences in the number of NMJs per section (N = 3 animals per genotype). C) Quantification of α-bungarotoxin labeled NMJs demonstrates no difference in total area (as measured by delineating outer edge and measuring area within that boundary, area analyzed is within white trace of 4Ai) of NMJs in *Cra1*/+ mice (N = 3 animals per genotype). D) NMJ complexity, as measured by the synaptic area of α-bungarotoxin label (4Aii), is significantly decreased in 2 month and 6 month old *Cra1*/+ mice (*P<0.05; N = 3 mice per genotype).

**Figure 5:**
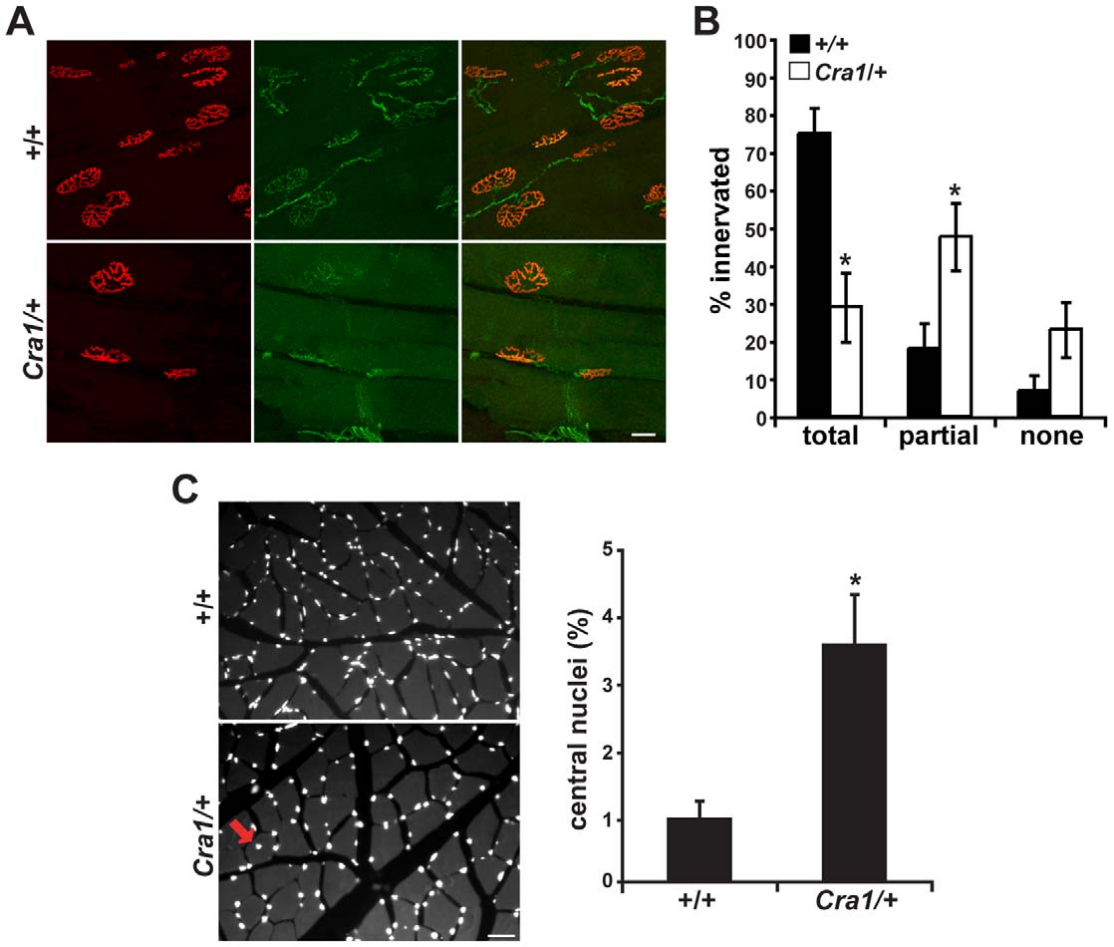
Synaptic and post-synaptic defects at the neuromuscular junctions in *Cra1/+* mice. A) α-bungarotoxin and GFP labeled NMJs. (Scale bar  = 20 µm). B) Motor neuron axons reveal a decrease in the proportion of NMJs with complete overlap between pre and post-synaptic zones and a compensatory increase in the proportion of NMJs with partial overlap in *Cra1/+* mice (*P<0.05). C) Labeling of nuclei by DAPI stain demonstrates a significant increase in central nuclei in *Cra1/+* mice, a measure of muscle fiber degeneration and regeneration (central nuclei indicated with red arrow; *P<0.05; N = 3 mice per genotype).

Although motor neuron cell bodies appear to be spared in symptomatic *Cra1*/+ animals, synaptic dysfunction at the neuromuscular junction (NMJ) could potentially underlie the observed behavioral abnormalities. This possibility would be consistent with genetic data from *Caenorhabditis elegans* and *Drosophila melanogaster*, where mutations in dynein components cause defects in synapse assembly [17]–[20]. Therefore, we examined neuromuscular junction synapses in 2 month and 6 month old *Cra1*/+ and *+/+* mice using α-bungarotoxin labeling of the tibialis anterior muscle (Figure 4A). In *Cra1*/+ mice, there is no reduction in the number of NMJs (Figure 4B). We examined the “total area” of each NMJ, defined by the area contained within a boundary encompassing the outermost edges of the NMJ, as revealed by α-bungarotoxin label (Figure 4Ai). There is no difference in NMJ total area between *Cra1/+* and *+/+* at 2 months and 6 months of age (Figure 4C). However, because of the complex, pretzel-like structure of the NMJ, total area contained within the outside boundary does not accurately reflect the “synaptic area” of pre- and post-synaptic alignment where intercellular communication occurs. Therefore, we also assessed the synaptic area by quantifying the area within each NMJ that is labeled by α-bungarotoxin (Figure 4Aii). The synaptic area within each NMJ is significantly reduced in *Cra1/+* mice at 2 and 6 months of age (Figure 4D) suggesting a reduction in complexity of the post-synaptic component. To assess the presynaptic zone of the NMJ synapse, we imaged motor neuron axons as they innervate the NMJ, using mice expressing GFP under the control of the HB9, motor neuron-specific promoter [21]. When we costained the NMJs with α-bungarotoxin and anti-GFP (Figure 5A), we found that there is decreased innervation of NMJs in *Cra1/+* mice (Figure 5B). Similar results were seen when we co-stained with α-bungarotoxin and neurofilament to visualize presynaptic motor neurons. Notably, quantitative analysis of presynaptic innervation of NMJs revealed that in *Cra1/+* animals, there are fewer NMJs that exhibit complete overlap of the presynaptic axon and the post-synaptic acetylcholine (ACh) receptors detected by α-bungarotoxin stain. Instead, the proportion of NMJs that exhibit partial overlap between the presynaptic axon and the post-synaptic ACh receptors is increased in mutant animals. Such partial overlap has also been observed in mouse models of spinal muscular atrophy [22], and suggests that there is instability of the presynaptic components of the NMJ. Together these data provide evidence of defects in both the pre and post-synaptic components of the mutant NMJs.

During adult life, muscle fibers can degenerate and be replaced by new muscle fibers; higher rates of muscle fiber turnover are seen in response to nerve degeneration or to NMJ dysfunction. As centrally localized nuclei mark the presence of regenerating muscle fibers [23], [21] we quantified centrally localized nuclei in the tibialis anterior muscles of 6 month old *Cra1*/+ and +/+ mice. Figure 5C demonstrates that there are more muscle fibers with central nuclei in *Cra1/+* animals, indicating increased muscle turnover. Such changes in muscle fiber could be a consequence of NMJ dysfunction, or might be a very early indicator of motor neuron axon degeneration and regeneration.

The results presented here suggest a novel mechanism to explain the behavioral abnormalities found in *Cra1*/+ mice. We find evidence of an early-onset, stable phenotype of hindlimb cramping and reduced grip strength. At symptomatic ages, we find reduced morphological complexity in the neuromuscular junction. This pathology occurs in the absence of either motor or sensory neuron loss, suggesting that a developmental synaptic dysfunction rather than an adult onset neurodegenerative syndrome underlies the behavioral deficits in *Cra1*/+ mice.

## Discussion

Previous reports on mice with hypomorphic mutations in dynein, the *Cra1*/+, *Loa/+* or *Swl/+* mice, described a late-onset degeneration of motor neurons, with progressive decline in motor neuron function and number [9], or an early-onset degeneration of proprioceptive sensory neurons. Our recurrent behavioral assessment of *Cra1*/+ mice shows an early onset, stable phenotype of hindlimb cramping and impaired grip strength, with no further progression in adulthood over the 24 months of testing. We examined the anatomical underpinnings of this behavior. We find no loss of either motor or sensory neurons at symptomatic ages; instead, our analysis identifies decreased complexity of neuromuscular junction synapses, with associated muscle fiber abnormalities.

Dynein and its associated dynactin complex have been shown to be important for synapse formation and stabilization in both *Drosophila* and *C. elegans* [17], [11], [19], [20]. In *C. elegans*, mutations in genes encoding components of the dynein motor, including dynein heavy chain, results in abnormal assembly of synapses. Additionally, these mutants exhibit intracellular protein aggregations and behavioral abnormalities including locomotor impairment, and shorter lifespan [19]. *Drosophila* with mutations in genes encoding components of the dynein/dynactin complex, as well as those with mutations in dynein light chain genes, exhibit defects in synapses of sensory and motor neurons [17]–[20]. These studies indicate that dynein functions in the presynaptic terminal to promote synapse stabilization and prevent synapse elimination [18].

Our findings of abnormal NMJ complexity in *Cra1*/+ mice suggests that dynein plays a role in development and maintenance of mammalian synapses as well. The morphological changes in NMJ synapses and the associated alterations in muscle fibers, as well as the early onset of behavioral deficits in *Cra1*/+ mice, suggest that synapse formation, stability and refinement are altered by defects in dynein function. Thus, the *Cra1*/+ mice provide a useful model to examine the role of dynein in the dynamic processes of NMJ synapse formation and stabilization.

It is currently unclear why there are such large discrepancies in the phenotypes of the *Cra1*/+ and *Loa*/+ mice as described by different groups. Clearly these mutations in the dynein heavy chain gene affect the lower motor neuron circuitry comprising the primary motor neurons, the innervated muscle and the proprioceptive neurons that innervate the muscles. It is apparent that deterioration in a particular portion of this circuitry can ultimately result in dysfunction of the entire circuit. However, it has not been clear where or how dynein mutations initiate abnormalities in this circuit.

Our behavioral analysis reveals that the mice exhibit deficits from an early age, while anatomical analysis indicates that disruption of NMJ synaptic structure is present and precedes any motor or sensory neuron loss. We did not observe any evidence of late degeneration; however, it is possible that early disruption in synaptic function at the NMJ could prime motor neuron or sensory neuron cell bodies for later degeneration under some conditions [11], [9], [12]. Thus, it may be important to consider the effect of environment on the manifestation of phenotypes in these mice. Recent evidence suggests that exercise and calorie intake can dramatically affect age-related changes in neuromuscular junctions in mice [24]. Since we find that one of the primary sites of dysfunction in *Cra1*/+ mice is at the NMJ, variations among laboratories in housing conditions and daily food intake will impact the emergence of this phenotype and may account for some of the divergent reports of phenotypes.

Intriguingly, a recent study by Braunstein et al. finds striatal atrophy, abnormal dopamine signaling, and impaired neurite outgrowth of striatal neurons in *Cra1*/+ mice. These data suggest that under some conditions dynein mutations may affect multiple circuits in the central as well as the peripheral nervous system [25].

In conclusion, we provide evidence for an early-onset, stable phenotype in *Cra1*/+ mice. In contrast to previous reports, we find no evidence of either motor or sensory neuron loss at symptomatic ages. Instead, we find evidence of altered topology of NMJs, in that the area within each synapse that is covered by acetylcholine receptors is reduced, together with a reduction in the region of overlap with the presynaptic axon terminal. We propose that disruption of dynein function in *Cra1*/+ mice results in abnormalities of neuromuscular junction synapse formation, refinement, and/or stabilization. Therefore, *Cra1*/+ mice may prove to be a useful tool to examine the process of synaptogenesis and maturation at the mammalian NMJ.

## Materials and Methods

### Animal Use

*Cra1*/+ mice were purchased under an MTA agreement from Ingenium Pharmaceuticals. Genotyping for the *Cra1* point mutation was performed by Transnetyx, Inc. (Cordova, TN) using a wild type or Cra1 targeting sequence (TGTGTTTCCTTTGCAGGTTTGGCTTCAGT**[A/G]**C CAGTGTTTGTGGGATATGCAGGCAGAAAACATTTACAACAGGCTAGGGGAA). HB9-GFP mice [21] were obtained from Jackson Laboratory. To obtain *Cra1*/+ mice expressing GFP in motor neurons, HB9-GFP heterozygous mice (HB9-GFP/+) were crossed with *Cra1*/+ mice to obtain *Cra1*/+ HB9-GFP/+ mice. Only doubly-heterozygous offspring from the original *Cra1*/+ and HB9-GFP/+ cross were used. All experimental procedures were done in accordance with the National Institutes of Health guidelines and were approved by the Dana-Farber Cancer Institutional Animal Care and Use Committee. The permit number is 03-054.

### Behavior Analyses

Kaplan-Meier survival curves were generated using Prism statistical software, and analyzed using the Curve Comparison analysis for survival data.

Cramping behavior was measured by lifting the animal gently by the tail, suspending the hindlimbs and leaving forelimbs touching the ground. Two independent observers scored the animal as 1 (no hindlimb abnormalities), 2 (cramping of hindlimbs), or 3 (paralysis of one or both hindlimbs). Scorers were blind to genotype.

Grip strength was measured using a digital grip strength meter, which records the maximal strength an animal exerts while trying to resist an opposing pulling force (Columbus Instruments, Columbus, OH). Forelimb grip strength was measured using a mouse tension bar, and hindlimb grip strength was measured using a mouse compression bar. The results of three consecutive trials on the same day were averaged for each animal. Significance was calculated by Student's two-tailed t-test. Statistical tests were performed at every third time point (3 months, 6 months, etc.). Testers were blind to genotype.

### Motor Neuron Labeling

*Cra1*/+ and +/+ mice were euthanized and perfused with PBS, followed by 4% paraformaldehyde (PFA). The lumbar enlargement of the spinal cord was removed, post-fixed in 4% PFA overnight at 4°C, then cryopreserved in 30% sucrose at 4°C overnight. Spinal cords were frozen at −20°C, and sliced at 14 µm. For ChAT staining, slices were blocked in 10% normal donkey serum and 0.1% TritonX-100 in PBS for 1 hour at room temperature, then incubated in goat anti-choline acetyltransferase (ChAT) antibody (1∶100, Chemicon, Cat# AB144P) overnight at 4°C. Tissue was then incubated in biotinlyated donkey anti-goat antibody (1∶300, Vector Laboratory), followed by streptavidin conjugated Alexa 546 (1∶500, Invitrogen), then counterstained with DAPI (1∶1000) for 1 minute. Images were obtained with a 40X oil objective using NIS Elements software. ChAT labeled motor neurons were quantified by counting neurons containing a nuclear profile; 8 sections of the spinal cord anterior horn per animal, from three sets (*Cra1*/+ and +/+) of animals at each age were analyzed. For toluidine blue staining, slices were dried on a hot plate and a drop of filtered toluidine blue solution (1% toluidine blue O (basic blue 17), 1% sodium borate) was added to the slide and then rinsed in water when the solution began to dry. Images were obtained with a 10X objective using NIS Elements software. Toluidine blue labeled motor neurons were quantified by counting only neurons greater than or equal to 600 µm^2^ in size; 8 sections of the spinal cord anterior horn per animal, from three pairs (one *Cra1*/+ and one +/+) of animals at each age were analyzed.

### Sensory Neuron Labeling

*Cra1*/+ and +/+ mice were euthanized and perfused with PBS, followed by 4% PFA. L4 and L5 dorsal root ganglia (DRG) were removed, post-fixed in 4% PFA overnight at 4°C, then crysostat sections along the longitudinal plane of the ganglia were prepared as described above. For parvalbumin and ER81 labeling, sections were blocked in 10% normal goat serum and 0.1% TritonX-100 in PBS for 1 hour at room temperature, then incubated in rabbit anti-parvalbumin (Abcam, 1∶200, Cat# ab45542) or rabbit anti-ER81 (Abcam, 1∶1000, Cat# ab36788) overnight at 4°C. Tissue was then incubated in goat anti-rabbit Alexa-546 (Molecular Probes, 1∶1000) for 1 hour at room temperature, and then counterstained with DAPI. For TrkC staining, sections were blocked in 10% normal horse serum and 0.1% TritonX-100 in PBS for 1 hour at room temperature, then incubated in goat anti-TrkC (R&D Systems, 1∶100, Cat# AF1404) overnight at 4°C. Tissue was then incubated in horse anti-goat Alexa-546 (Molecular Probes, 1∶1000) for 1 hour at room temperature, and then counterstained with DAPI.

Proprioceptive sensory neurons were quantified by counting 8 sections per animal, from three sets (*Cra1*/+ and +/+) of animals. Number of labeled neurons containing a nucleus were counted, and compared to the total number of nuclei in the section to calculate the percentage of sensory neurons that are proprioceptors.

For labeling of proprioceptive fibers within the spinal cord, *Cra1*/+ and +/+ mice were euthanized and perfused with PBS, followed by 4% PFA, and sections of the lumbar enlargement of the spinal cord were prepared as described above. Sections were blocked in 10% normal goat serum and 0.1% TritonX-100 in PBS for 1 hour at room temperature, then incubated in rabbit anti-parvalbumin (Swant, 1∶1000, Cat# PV-28) overnight at 4°C. Tissue was then incubated in goat anti-rabbit Alexa-546 (Molecular Probes, 1∶1000) for 1 hour at room temperature, and then counterstained with DAPI. Three pairs (one *Cra1*/+ and one +/+) of animals were examined.

### Neuromuscular Junction Labeling

*Cra1*/+ and +/+ mice were euthanized and perfused with PBS, followed by 4% PFA. The tibialis anterior muscle was removed, post-fixed in 4% PFA overnight at 4°C, then cryopreserved in 30% sucrose at 4°C overnight. Muscles were frozen at −20°C, and sliced longitudinally at 40 µm. Muscle tissue was blocked in 3% bovine serum albumin, 2.5% normal goat serum, and 0.1% TritonX-100 in PBS for 1 hour at room temperature, then incubated in α-bungarotoxin-tetramethylrhodamine (1∶500, Molecular Probes, Cat# T1175) overnight at 4°C, then counterstained with DAPI. Confocal images were obtained using a Zeiss LSM 510 META upright confocal microscope, using a 40X air objective.

Measurements of overall NMJ area were determined by delineating the outside edge of the α-bungarotoxin labeled NMJ, then measuring the total area within that outline. For each synapse, measurements of complexity were obtained by measuring the area of fluorescent signal that is above background and is within the outline of the NMJ. NMJ analysis was done using Metamorph software by counting 8 sections of the tibialis anterior muscle per animal from three pairs (one *Cra1*/+ and one +/+) of animals at each age.

### Muscle Fiber Labeling

Cryostat sections of the tibialis anterior muscle from *Cra1*/+ and +/+ mice were prepared and stained as above. Centrally localized nuclei were counted and compared to the total number of nuclei in the section to calculate the percentage regenerating muscle fibers. Quantification was done by using 8 sections of the tibialis anterior muscle per animal from three pairs (one *Cra1*/+ and one +/+) of animals.
